# The Association between Echocardiographic Parameters of Heart Failure with Preserved Ejection Fraction and Fluid Status Biomarkers in Hemodialysis Patients

**DOI:** 10.3390/diagnostics14121310

**Published:** 2024-06-20

**Authors:** Mariusz Lupa, Agnieszka Pardała, Anna Bednarek, Jolanta Mrochem-Kwarciak, Regina Deja, Katarzyna Mizia-Stec, Aureliusz Kolonko

**Affiliations:** 1 Department of Internal Medicine, District Hospital, 34-600 Limanowa, Poland; mariuszlupa@poczta.onet.pl; 2 Dialysis Centre, Fresenius Nephrocare, 34-600 Limanowa, Poland; kaniaa@mp.pl; 3 First Department of Cardiology, Medical University of Silesia, 40-635 Katowice, Poland; annabednarekmd@gmail.com (A.B.); kmizia-stec@sum.edu.pl (K.M.-S.); 4 Analytics and Clinical Biochemistry Department, Maria Skłodowska-Curie National Research Institute of Oncology, 44-102 Gliwice, Poland; jolanta.mrochem-kwarciak@io.gliwice.pl (J.M.-K.); regina.deja@nio.gov.pl (R.D.); 5 Department of Nephrology, Transplantation and Internal Medicine, Medical University of Silesia, 40-027 Katowice, Poland

**Keywords:** copeptin, diastolic dysfunction, heart failure, lung comets, N-terminal prohormone for brain natriuretic peptide, mid-regional pro-atrial natriuretic peptide

## Abstract

Overhydration and cardiac function abnormalities are common in hemodialysis patients. The association of N-terminal prohormone for brain natriuretic peptide (NT-proBNP) and other fluid status biomarkers with echocardiographic parameters of heart failure with preserved ejection fraction (HFpEF) is scarcely investigated in this population. A total of 100 separate measurements performed in 50 dialysis patients (29 male, aged 60 ± 17 years) in NYHA class II/II and preserved left ventricle ejection fraction were analyzed. Plasma levels of NT-proBNP, mid-regional prohormone for atrial natriuretic peptide (MR-proANP) and copeptin (CPP) were measured. The E/e’ ratio as an index of HFpEF and other echocardiographic parameters were calculated. An E/e’ ratio >9 was associated with higher median right ventricular systolic pressure (RVSP) and LVMI values. Left atrium volume index (LAVI) as well as NT-proBNP and MR-proANP, but not CPP levels were significantly higher in this group. In a stepwise multivariate analysis, only CPP and IL-6 levels were found to be independently associated with the E/e’ ratio in the study group, whereas NT-proBNP and MR-proANP were associated only with left heart structure parameters and LVEF. Of the analyzed biomarkers, only the CPP level was found to be independently associated with the E/e’ ratio in maintenance hemodialysis patients.

## 1. Introduction

In chronic kidney disease (CKD) patients treated with hemodialysis (HD), hydration status assessment and adequate fluid control remains one of the most challenging clinical problems. Persistent hypervolemia results in difficult-to-treat hypertension and increased arterial stiffness, followed by complex cardiac damage, including left ventricular hypertrophy (LVH) and chronic heart failure [1,2,3]. Moreover, the negative consequences of long-term pre-transplant fluid overload also remain important in the post-transplant period, causing the increased all-cause and cardiovascular mortality of kidney transplant recipients [4]. Therefore, several measures of hydration status are currently used in the hemodialysis population in an effort to precisely set the patients’ dry weight and to optimize the individual ultrafiltration prescription. They include a clinical assessment, different ultrasound-based measurements (inferior vena cava diameter, echocardiography, number of lung comets), bioimpedance analysis, and circulating biomarkers assessment [5,6].

Among the various cardiac abnormalities found in CKD patients, left ventricular (LV) diastolic dysfunction is one of the most prevalent [7]. The structural changes which occur in the heart in uremic cardiomyopathy include LVH, myocardial fibrosis, and impaired coronary microcirculation [8,9]. Those entities, together with elevated inflammatory markers and worse nutritional status [10], result in the development of diastolic dysfunction and heart failure with preserved ejection fraction (HFpEF). This condition was suggested to be a risk factor for CKD progression [11], whereas in HD patients it was proportionally associated with mortality [7,12] and cardiovascular events [12]. On the other hand, the recommended diagnostic process of HFpEF based on symptoms (±signs), increased levels of natriuretic peptides and/or echocardiographic parameters (ESC) has significant limitations in dialysis patients. Overhydration measured as an amount of extracellular water was demonstrated to be independently associated with E/e’ ratio and LVMI in pre-dialysis subjects [2,13]. However, the association of N-terminal prohormone for brain natriuretic peptide (NT-proBNP) and other fluid status biomarkers blood levels with echocardiographic parameters of HFpEF in hemodialysis patients has rarely been investigated. Thus, in the present prospective study, we chose the E/e’ ratio as a reference parameter for the diagnosis of HFpEF in hemodialysis patients and aimed to analyze its relationship with other hypervolemia and raised LV filling pressure markers, including several natriuretic hormones concentrations. The New York Heart Association (NYHA) Functional Classification was used in this study. Each NYHA class describes the patient’s symptoms at rest and while performing daily physical activities, allowing an individual staging of heart performance, from class I (without any limitations of physical activity) to class IV (when symptoms of heart failure are present at rest).

## 2. Materials and Methods

### 2.1. Study Population

This cross-sectional study included 50 patients with chronic kidney disease (CKD), in NYHA class II or III, with LV ejection fraction (LVEF) ≥50%, who received hemodialysis treatment 3 times a week. The study was performed between July 2015 and October 2017. Patients with atrial fibrillation, active infection, hepatic cirrhosis, and cancer were excluded from the study. The study protocol was accepted by the Bioethics Committee of the Medical University of Silesia in Katowice (KNW/0022/KB1/47/14), and all participants provided written informed consent. The study was conducted in accordance with the Declaration of Helsinki.

In each patient, 2 sets of measurements were performed: immediately prior to the first and third dialysis session in the same calendar week, mainly due to the commonly observed difference in the degree of hypervolemia, which is a consequence of different numbers of days (2 or 3) from the last dialysis session. Both investigations comprised echocardiography and chest ultrasound. Additionally, blood samples were withdrawn at the same time-points. Patients were analyzed as a total group and in the subgroups divided according to the E/e’ ratio: ≤ and >9.

### 2.2. Clinical and Anthropometric Measurements

Body weight and height were measured following standard procedures, and body mass index (BMI) was calculated (kg/m^2^). Body surface area (BSA) was calculated according to the DuBois formula [0.20247 × weight (kg)^0.425^ × height (m)^0.725^] and expressed in m^2^.

Blood pressure was measured during the patients’ examination immediately before each dialysis session.

### 2.3. Laboratory Measurements

Biochemical parameters, including serum albumin, hemoglobin concentration, and high-sensitivity C-reactive protein (hs-CRP) level were measured using standard laboratory methods.

Plasma levels of IL-6 (R&D Systems, Minnesota, MN, USA) were assessed by ELISA, with the intra-assay and inter-assay coefficients of variability <7.2 and <7.8%, respectively. Plasma NT-proBNP concentration was measured by the electrochemiluminescence method using a commercially available Cobas E411 analyzer (Roche Diagnostics GmbH, Mannheim, Germany) with intermediate precision < 4.6%. Plasma concentrations of copeptin (C-terminal precursor for pro-vasopressin, CPP) and mid-regional pro-atrial natriuretic peptide (MR-proANP) were measured with the reference method B.R.A.H.M.S using, respectively, KRYPTOR and KRYPTOR II analyzers (Thermo Fisher Scientific Inc., Waltham, MA, USA).

### 2.4. Echocardiography

Echocardiographic measurements were performed using Hitachi Aloka ProSound Alpha 6, equipped with 1–15 MHz cardio transducer UST 5299 (Tokyo, Japan). Full-mode and two-dimensional measurements were performed as recommended by the American Society of Echocardiography [14] by one experienced echocardiographer. The measurements included left ventricular end-diastolic (LVEDD) and end-systolic (LVESD) diameters, intraventricular septum (IVS), and posterior wall (PW) end-diastolic thickness. LVEF was calculated by using the biplane Simpson method. Left (LA) and right atrium (RA) volumes were calculated according to the Simpson method. Right ventricular systolic pressure (RVSP), a surrogate for pulmonary artery systolic pressure (PASP), was calculated from the apical 4-chamber view as a measured velocity of tricuspid backflow wave with added 5–15 mmHg (range for mean right atrial pressure) [15]. Left ventricular mass (LVM) was calculated according to the Devereux formula [16] and it was indexed to the body surface area (BSA). To record the trans-mitral blood flow at the apical four-chamber view, pulsed-wave Doppler was used. The rate of the E wave, the rate of the late diastolic blood flow (mitral peak velocity of late filling, A), and the ratio of E/A were measured. In the septum and the lateral annulus, estimated left ventricular filling pressure via e’ was measured by tissue Doppler, and the E/e’ ratio was calculated [12].

The frequency of abnormal values of the parameters involved in the definition of HFpEF by ESC was assessed [17].

### 2.5. Lung Ultrasound

Lung ultrasound examinations were performed using a Toshiba Xario machine equipped with a 3.5 MHz convex transducer. Patients were in a supine position. The probe was placed along the intercostal spaces (II–V on the right side and II–IV on the left side) in mid-axillary, anterior axillary, mid-clavicular, and parasternal lines. The number of B-lines was assessed for all locations and the sum was calculated for a given patient, denoting the extent of extravascular fluid in the lungs. At each scanning site, B-lines were counted from zero to ten, with zero indicating a complete absence of B-lines and a full white screen corresponding to ten B-lines [18].

### 2.6. Statistical Analysis

Statistical analyses were performed using the STATISTICA 13.3 PL for Windows software package (StatSoft Polska, Kraków, Poland) and MedCalc 20.014. (Mariakerke, Belgium). The values were presented as medians with first and third quartile values, or frequencies. Data were analyzed including all 100 separate measurements of clinical, laboratory, and imaging assessments in 50 study subjects. The main study comparison was performed between 2 groups, defined based on the value of mitral E/e’ ratio ≤ or >9, using Mann–Whitney U test or χ^2^ test, as appropriate. Correlation coefficients were calculated using the Spearman rank-test. The associations between study biomarkers and several echo parameters used to diagnose left heart dysfunction and the number of lung comets, as well as the associations between echo parameters and lung comets, were analyzed. Stepwise multivariate regression analyses were performed separately for the NT-proBNP, MR-proANP and CPP concentrations as dependent variables. The models were built based on univariate analyses and included all potential explanatory variables with *p* value < 0.1. In all statistical tests, ‘*p*’ values below 0.05 were considered statistically significant.

## 3. Results

### 3.1. Study Group

The study group consisted of 50 patients (29 male, 58.0%) with mean age of 60 ± 17 years and mean BMI, 25.4 ± 5.4 kg/m^2^. The median time of hemodialysis therapy was 33 ± 58 months. The primary cause of renal disease was glomerulonephritis (26.8%), hypertensive (16.1%) or diabetic (8.9%) nephropathy, autosomal dominant polycystic kidney disease (12.5%), pyelonephritis (21.4%), and other and unknown causes (14.3%). The major comorbidities included diabetes mellitus (28.6%), ischemic heart disease (60.7%), and hypertension (91.1%). Major adverse cardiovascular events, including stroke, myocardial infarction, and coronary artery stenting or bypass graft, were previously diagnosed in 26.0% of study participants.

### 3.2. The Frequency of Diastolic Left Ventricular Function Assessed Using Different Echo Criteria

The percentages of study measurements which fulfilled different specific criteria of diastolic heart dysfunction according to the ref. [17] are presented in Table 1.

### 3.3. Comparison of the Study Subgroups Assessments with E/e’ Value ≤ or >9

Among all the study measurements, in 72 (72%) the E/e’ index was ≤9 and in 28 (28%) was >9. There were no significant differences in age, sex, BMI, dialysis vintage, the amount of residual diuresis, major comorbidity, or NYHA class between those two subgroups (Table 2). Interestingly, in the E/e’ >9 subgroup, more than half of all measurements was performed in patients dialyzed using a venous catheter in contrast to the dominance of arterio-venous fistula access in the E/e’ ≤9 subgroup (Table 2). The median LVMI and LAVI were significantly greater in the higher E/e’ subgroup. This subgroup was also characterized by greater occurrence of lung comets. The values of both MR-proANP and NT-proBNP were significantly higher in the E/e’ >9 subgroup, whereas the CPP levels were comparable (Table 2). Notably, the value of E/A ratio was similar in both of the analyzed subgroups.

### 3.4. The Associations of Echo Parameters and Study Biomarkers with Lung Comets

The majority of the examined echo parameters of left ventricular dysfunction (except LVMI and RWT) were positively associated with the number of lung comets (Table 3).

When comparing the values of analyzed biomarkers with the measures of fluid status, we noted the significant associations of both NT-proBNP and MR-proANP with the number of pre-dialytic lung comets (R = 0.506; *p* < 0.001 and R = 0.250; *p* < 0.05, respectively) and LAVI (R = 0.575 and R = 0.531; both *p* < 0.001), whereas CPP showed no associations with abovementioned ultrasound-derived parameters (Table 3).

The NT-proBNP values strongly correlated with MR-proANP (R = 0.801; *p* < 0.001), but not with CPP levels (R = 0.047; *p* = 0.65). CPP was also not associated with MR-proANP values (R = 0.168; *p* = 0.09).

### 3.5. The Associations between Study Biomarkers and Echo Parameters

The associations between study biomarkers and the echo-derived parameters of left ventricular dysfunction are shown in Table 3. In the whole study group, both MR-proANP and NT-proBNP were positively associated with mean E/e’ (R = 0.370 and R = 0.511, respectively, both *p* values < 0.001). In contrast, there were only weak reverse correlations of CPP values with mean E/e’ value (R = −0.243; *p* < 0.05). Similarly, both NT-proBNP and MR-proANP significantly correlated with all echo parameters used in the diagnostics of left heart dysfunction, except RWT and pulmonary artery systolic pressure, whereas CPP did not correlate with any echo parameter except E/e’ (Table 3).

Interestingly, NT-proBNP, but not MR-proANP, was associated with age (R = 0.312; *p* < 0.01), residual diuresis (R = −0.222; *p* < 0.05), and serum albumin (R = −0.353; *p* < 0.001), whereas CPP was associated with residual diuresis (R = −0.228; *p* < 0.05) and serum albumin (R = 0.225; *p* < 0.05), but not with age. Both NT-proBNP and MR-proANP were associated with dialysis vintage (R = 0.317 and R = 0.312, respectively; both *p* < 0.01). On the other hand, only CPP (R = 0.257; *p* < 0.01), but not NT-proBNP or MR-proANP, was associated with mean whole-week ultrafiltration rate expressed as a percent of the patient’s dry body mass. No correlations were found regarding pre-dialysis SBP, DBP, and delta SBP during dialysis session, mean MAP value calculated during the preceding 6-month period, and normovolemic body weight as defined using bioimpedance analysis.

The results of univariate analyses for NT-proBNP, MR-proANP, and CPP values are presented in Table 4.

Stepwise multivariate regression analyses for the variability of all above biomarkers revealed that LVMI (r_partial_ = 0.371) and LVEF (r_partial_ = −0.458; both *p* < 0.001) were independently associated with NT-proBNP concentration (R^2^ = 0.37), whereas LVMI (r_partial_ = 0.231; *p* < 0.05), LVEF (r_partial_ = −0.221; *p* < 0.05) and LAVI (r_partial_ = 0.258; *p* < 0.01) independently influenced MR-proANP levels (R^2^ = 0.26). In contrast, E/e’ values (r_partial_ = −0.233; *p* < 0.05) and IL-6 levels (r_partial_ = −0.237; *p* < 0.05) were the only factors which influenced CPP concentration (R^2^ = 0.10).

During the follow-up period of 58 IQR, 27–71) months, 12 patients (70.6%) in the E/e’ > 9 and 10 patients (30.3%) in the E/e’ ≤ 9 died (*p* < 0.01). There was no statistical difference in MACE occurrence in both groups (*p* = 0.77).

## 4. Discussion

In the present study, we show biochemical and echocardiographic markers of hemodialysis patients with suspected HFpEF. The study population presented symptoms in NYHA class II or III and had preserved LVEF. Substantial discrepancies were found in the prevalence of echocardiographic parameters of HFpEF—the parameters of cardiac structural and/or functional abnormalities consistent with the presence of LV diastolic dysfunction/raised LV filling pressures. The frequency of abnormal values of these parameters was the highest in regard to the LVMI and RWT; and E/e’ index > 9 was found only in 28% of patients. The reciprocal relationships between three analyzed hormonal biomarkers (NT-proBNP, MR-proANP and copeptin) and several fluid status measures were also analyzed, as well as the profiles of different clinical risk factors for all natriuretic peptides studied. In multivariate analyses, only CPP and IL-6 levels were found to be independently associated with the E/e’ ratio in the study group, whereas NT-proBNP and MR-proANP were associated only with left heart structure parameters and LVEF.

The pathogenesis of LV diastolic dysfunction in CKD patients includes abnormal ventricular filling in diastole and a higher left ventricular filling pressure because of LVH, in addition to myocardial interstitial fibrosis [19]. As a consequence, this condition is commonly found in pre-dialysis CKD subjects, even with preserved systolic function [20], as well as in hemodialysis patients [21]. Importantly, the prevalence of diagnosed diastolic dysfunction differed significantly as it was dependent on the older (2009) or newer (2016) ASE/EACVI criteria used [20,22]. However, as volume overload and dyspnea symptoms, used in the cornerstone NYHA classification, are more commonly seen in CKD population, new, CKD-tailored criteria for heart failure diagnosis were proposed by the Acute Dialysis Quality Initiative (ADQI) a decade ago [23]. Later, Untersteller et al. noted that in a CKD stage G2–G4 cohort, those echocardiographic criteria are virtually omnipresent among CKD patients [24]. Notably, the E/e’ ratio is still used in the current research as a simple reference measure of diastolic heart dysfunction [21,24,25,26].

In the present study, we evaluated the percentages of different criteria of HFpEF, as described in the current ESC guidelines [17]. We found huge discrepancies regarding the frequency of abnormal values of these parameters, with the highest rate of the LVMI, RWT, and NT-proBNP criteria. NT-proBNP was elevated in all subjects, however, it cannot be used a priori for heart failure diagnosis in the dialysis population. The increased LVMI and RWT were probably consequences of the systemic hypertension. As all those entities are commonly and enormously elevated in the majority of hemodialysis patients, this suggests that they should be taken into account with caution during the diagnosis of HFpEF in this population. Analysis of the E/e’ index as a well-documented and relatively highly specific marker of HFpEF revealed that its values increased only in 28% of patients. Thus, we suspect that NYHA II/III class in our hemodialysis patients corresponded with HFpEF only in some of the patients.

In our study, we analyzed NT-pro-BNP, MR-proBNP, and copeptin as hormonal fluid status markers in the context of LV diastolic dysfunction identification. However, in multivariate analyses, which also included relevant inflammatory marker (IL-6), the number of lung comets, echocardiographic parameters (LVMI, LVEF, LAVI, E/e’ ratio), and other parameters revealed in univariate analyses (age, type of vascular access, previous cardiovascular disease, and serum albumin), only CPP and IL-6 levels were found to be independently associated with the E/e’ ratio in the study group, whereas NT-proBNP and MR-proANP were associated only with left heart structure parameters and LVEF. Instead, it was not associated with several measures of overhydration. CPP, a surrogate marker for proarginine vasopressin, has been reported to have prognostic value in chronic heart failure patients [27]. However, it failed to differentiate subjects with acute heart failure, including diastolic dysfunction, in other analyses [28,29]. In hemodialysis patients, the CPP level was found to correlate with pre-dialysis fluid volume, measured by bioimpedance analysis, and was higher in subjects with LV dysfunction [30]. Our previous research also suggested the possible dysregulation of physiological mechanisms of CPP secretion in hemodialysis patients, mostly due to permanent hyperosmolality and neurohormonal activation [31]. Nevertheless, to the best of our knowledge, this is the first analysis of CPP levels in the aspect of HFpEF in hemodialysis patients.

The main limitation of the present study is the restricted number of patients. However, this prospective analysis was performed using several measures of patient’s fluid status, assessed by the same physician, and the determination of all study hormonal biomarkers was performed using the reference laboratory methods, in contrast to other commercially available kits, which yielded inconclusive results in our study group. We have no data on the inter- and intra-observer variability of echocardiographic measurements; however, the study was performed by one experienced echocardiographer.

## 5. Conclusions

The copeptin level was found to be independently associated with the E/e’ ratio in hemodialysis patients, whereas NT-proBNP and MR-proANP were associated only with left heart structure parameters and LVEF. As the LVMI, RWT, and NT-pro-BNP are commonly and enormously elevated in the majority of hemodialysis patients, this suggests that they should be taken into account with caution during the diagnosis of HFpEF in this population. Nevertheless, as both copeptin and MR-proANP measurements are not easily affordable in many hospitals, it should be stressed that echocardiography and lung sonography are widely available tools which could be very useful in determining the fluid status in hemodialysis patients in daily clinical practice.

## Figures and Tables

**Table 1:** The percentage of study measurements consistent with the diagnosis of diastolic heart dysfunction.

Parameter	Percentage of Diastolic Heart Dysfunction
LV mass index ≥ 95 g/m^2^ (female) or ≥115 g/m^2^ (male)	88.0%
RWT > 0.42	70.0%
LA volume index > 34 mL/m^2^	39.0%
E/e’ > 9	28.0%
NT-proBNP > 365 pg/ml	100%
PA systolic pressure > 35 mmHg	51%
TR velocity at rest > 2.8 m/s	13.4%

LV—left ventricle; RWT—relative wall thickness; LA—left atrium; NT-proBNP—N-terminal prohormone of brain natriuretic peptide; PA—pulmonary artery; TR—tricuspid regurgitation.

**Table 2:** The demographic and clinical characteristics with ultrasound measurements in study subgroups, based on the value of E/e’ ratio ≤ 9 or >9.

Parameter	E/e’ ≤ 9	E/e’ > 9	*p*
	72 Measurements	28 Measurements	
Age [years]	62 (55–69)	67 (54–74)	0.26
Sex [M/F]	45/27	13/15	0.14
BMI [kg/m^2^]	25.4 (20.8–30.0)	24.9 (20.5–29.6)	0.25
Dialysis vintage [months]	32 (7–66)	41 (7–73)	0.94
Vascular access			<0.001
AVF [n, %]	50 [68.4]	14 [48.5]	
Catheter [n, %]	22 [31.6]	14 [51.5]	
Residual diuresis [mL]	500 (200–1000)	500 (225–1000)	0.74
Smoking status [%]	6.9 5/67	10.7 3/25	0.53
Hypertension [%]	87.5 63/9	96.4 27/1	0.18
Diabetes [%]	26.4 19/53	32.1 9/19	0.57
CVD [n, %]	52.8 38/34	71.4 20/8	0.09
NYHA class [n, %]			0.50
II	77.8 56/16	71.4 20/8	
III	22.2 16/56	28.6 8/20	
Albumin [g/dL]	4.0 (3.8–4.2)	3.8 (3.6–4.0)	<0.01
hs-CRP [mg/L]	6.4 (2.6–20.0)	4.9 (2.6–15.2)	0.39
IL-6 [pg/mL]	4.1 (2.2–9.0)	5.0 (2.8–12.7)	0.28
LVMI	147 (125–167)	169 (124–213)	<0.05
LVEF [%]	60 (55–65)	58 (50–65)	0.71
RVSP [mmHg]	32 (29–39)	38 (34–45)	<0.01
LAVI [mL/m^2^]	28.8 (20.7–34.8)	42.6 (32.2–53.5)	<0.001
RAVI [mL/m^2^]	18.0 (14.0–22.7)	18.3 (15.8–28.1)	0.47
IVRT [ms]	102 (90–132)	102 (72–129)	0.18
E/A	1.05 (0.80–1.30)	0.98 (0.73–1.10)	0.62
E/e’	6.9 (5.7–7.9)	10.1 (9.4–12.8)	<0.001
LUTs [n]	7 (3–12)	12 (7–41)	<0.001
NT-proBNP [pg/mL]	2642 (1551–5315)	5399 (3409–16,441)	<0.001
MR-proANP [pmol/L]	577 (455–748)	770 (610–1120)	<0.01
CPP [pmol/L]	122 (69–190)	98 (39–153)	0.12

Data presented as medians with Q1-Q3 values or frequencies. BMI—body mass index; AVF—arterio-venous fistula; CVD—cardio- and cerebrovascular disease; NYHA—New York Heart Association class; hs-CRP—high-sensitivity C-reactive protein; IL-6—interleukin 6; LVMI—left ventricular mass index; LVEF—left ventricular ejection fraction; RVSP—right ventricular systolic pressure; LAVI—left atrial volume index; RAVI—right atrial volume index; IVRT—isovolumic relaxation time; LUTs—lung comets; NT-proBNP—N-terminal prohormone for brain natriuretic peptide; MR-proANP—mid-regional pro-atrial natriuretic peptide; CPP—copeptin.

**Table 3:** The associations of echo parameters of diastolic heart dysfunction with study biomarkers and the number of lung comets.

Parameter	NT-proBNP [pg/mL]	*p*	MR-proANP [pmol/L]	*p*	CPP [pmol/L]	*p*	LUTs [n]	*p*
LVMI [g/m^2^]	0.264 (0.071–0.437)	<0.01	0.314 (0.125–0.481)	<0.01	−0.172 (−0.356–0.026)	0.09	0.073 (−0.125–0.266)	0.47
RWT	0.026 (−0.171–0.221)	0.80	−0.054 (−0.248–0.144)	0.59	−0.070 (−0.263–0.128)	0.49	0.108 (−0.090–0.298)	0.28
LAVI [ml/m^2^]	0.575 (0.427–0.693)	<0.001	0.531 (0.373–0.659)	<0.001	−0.079 (−0.271–0.120)	0.44	0.317 (0.129–0.484)	<0.01
Mean E/e’	0.511 (0.350–0.643)	<0.001	0.370 (0.187–0.528)	<0.001	−0.243 (−0.419–−0.049)	<0.05	0.388 (0.208–0.543)	<0.001
NT-proBNP [pg/mL]	-	-	0.801 (0.718–0.862)	<0.001	0.047 (−0.151–0.241)	0.65	0.506 (0.344–0.639)	<0.001
PA systolic pressure [mmHg]	0.272 (0.080–0.445)	<0.01	0.169 (−0.028–0.354)	0.10	−0.129 (−317–0.070)	0.20	0.323 (0.135–0.488)	<0.001
TR velocity at rest [m/s]	0.312 (0.123–0.479)	<0.01	0.233 (0.039–0.411)	<0.05	−0.180 (−0.363–0.017)	0.07	0.320 (0.132–0.486)	<0.01
LUTs [n]	0.506 (0.344–0.639)	<0.001	0.250 (0.057–0.426)	<0.05	−0.194 (−0.376–0.003)	0.06	-	-

NT-proBNP—N-terminal prohormone of brain natriuretic peptide; MR-proANP—mid-regional pro-atrial natriuretic peptide; CPP—copeptin; LUTs—lung comets; LVMI—left ventricle mass index; RWT—relative wall thickness; LAVI—left atrium volume index; PA—pulmonary artery; TR—tricuspid regurgitation.

**Table 4:** Results of univariate analyses.

	NT-proBNP			MR-proANP			CPP		
	β	95% CI	*p*	β	95% CI	*p*	β	95% CI	*p*
Age	97.5	−128.5–223.5	0.13	2.6	−1.6–6.8	0.23	−1.2	−2.1–−0.2	<0.05
Vascular access type (catheter vs. AVF)	−427	−4931–4079	0.85	−72.8	−222.2–76.6	0.34	−37.2	−71.2–3.2	<0.05
Previous CVD	6701	1957–11446	<0.01	66.4	−97.3–230.1	0.42	−35.2	−72.6–2.2	0.07
NYHA class	2643	−2393–7679	0.30	86.6	−81.2–254.5	0.31	−20.0	−58.8–19.0	0.31
LVMI	133.6	95.3–171.9	<0.001	3.7	2.3–5.1	<0.001	−0.3	−0.6–0.1	0.15
LVEF	−660.1	−1010.5–310.9	<0.001	−10.7	−22.9–1.6	0.09	3.3	0.5–6.1	<0.05
LAVI	303.4	154.7–452.1	<0.001	9.9	4.9–14.9	<0.001	−0.3	−1.5–1.0	0.65
LUTs	50.9	−28.2–130.0	0.20	1.7	0.93–4.3	0.20	−0.8	−1.4–−0.2	<0.05
E/e’	614.0	3.3–1224.7	<0.05	22.8	2.6–43.0	<0.05	−6.6	−11.3–−2.0	<0.01
Serum albumin	−451.3	−895.2–−7.5	<0.05	−15.4	−30.1–−0.7	<0.05	3.7	−0.2–7.0	<0.05
IL-6	385.1	56.2–714.1	<0.05	5.2	−6.0–16.4	0.36	−3.3	−5.9–−0.8	<0.01

NT-proBNP—N-terminal prohormone for brain natriuretic peptide; MR-proANP—mid-regional pro-atrial natriuretic peptide; CPP—copeptin; AVF—arterio-venous fistula; CVD—cardio- and cerebrovascular disease; NYHA—New York Heart Association class; LVMI—left ventricular mass index; LVEF—left ventricular ejection fraction; LAVI—left atrial volume index; LUTs—lung comets; IL-6—interleukin 6.

## Data Availability

The data presented in this study are available on reasonable request from the corresponding author.
